# Heparin Saline Versus Normal Saline for Flushing and Locking Peripheral Venous Catheters in Decompensated Liver Cirrhosis Patients: A Randomized Controlled Trial

**DOI:** 10.1097/MD.0000000000001292

**Published:** 2015-08-07

**Authors:** Rui Wang, Ming-Guang Zhang, Ou Luo, Liu He, Jia-Xin Li, Yun-Jing Tang, Yan-Li Luo, Min Zhou, Li Tang, Zong-Xia Zhang, Hao Wu, Xin-Zu Chen

**Affiliations:** From the Department of Gastroenterology (RW, OL, LH, MZ, LT, Z-XZ, M-GZ, HW); Infusion Nursing IV Team, West China Hospital, Sichuan University, Chengdu, China (RW, Y-LL); Faculty of Medicine, West China School of Medicine (Y-JT); Department of Hepatic Surgery (Y-LL); Department of Gastrointestinal Surgery, West China Hospital, Sichuan University, Chengdu, China (X-ZC).

## Abstract

A prospective randomized, controlled, single-blinded trial to compare the effectiveness and safety of heparin saline (HS) to those of normal saline (NS) as flushing and locking solutions for peripheral venous catheter (PVC) in decompensated liver cirrhosis (DLC) patients.

Patients with DLC at our institution between April 2012 and March 2013 were enrolled after obtaining informed consent. The patients were randomly allocated into 2 groups: the NS group received preservative-free 0.9% sodium chloride as the flushing and locking solution, while the HS group received HS (50 U/mL). PVC-related events and the duration of PVC maintenance were compared between the 2 groups. Moreover, the preinfusion and postinfusion levels of prothrombin time (PT), activated partial thromboplastin time (APTT), and platelet (PLT) were also compared.

A total of 32 and 36 DLC patients in the NS (125 PVCs) and HS (65 PVCs) groups, respectively, were analyzed. Baseline characteristics, including gender, age, Child–Pugh grade, PVC type and administration of anticoagulant, and irritant agents, were comparable between the 2 groups (*P* > 0.05). The maintenance times of the HS and NS groups were 80.27 ± 26.47 and 84.19 ± 29.32 hours, respectively (*P* = 0.397). Removal of PVC for abnormal reasons occurred in 30.7% and 22.4% of patients in the HS and NS groups (*P* = 0.208). The PVC occlusion rates were 6.2% and 5.6% in the HS and NS groups, respectively (OR = 1.11, 95% CI 0.31–3.92). The PT, APTT, and PLT levels were comparable between the 2 groups both before and after infusion (*P* > 0.05). Incremental analyses showed that Child–Pugh grade C might be a risk factor for the suppression of PLT in the HS group.

We consider NS to be as effective as and safer than conventional HS for flushing and locking PVC in decompensated liver cirrhosis patients.

## Abstract 2

Supplemental Digital Content is available in the text

## INTRODUCTION

As peripheral venous catheter (PVC) has been widely used in infusion treatment, the importance of flushing and locking techniques for PVC has received great attention.^1,2^ The most commonly used flushing and locking solution in China is heparin saline (HS), at a concentration of 10–100 U/mL previously.^3^ However, HS can cause thrombocytopenia when it is used to flush and lock catheters.^4,5^ Additionally, manual preparation of HS has a contamination risk of 8%–10%.^6,7^

Decompensated liver cirrhosis (DLC) patients can experience various pathophysiological changes, such as hepatic dysfunction, decreased synthesis of coagulation factors, hypersplenism, and increased capillary fragility.^8,9^ Therefore, these patients have an increased bleeding tendency.^9^ Heparin is a highly sulfated glycosaminoglycan with the highest negative charge density of any known biological molecule; it is widely used as an injectable anticoagulant and can also be used to form an inner anticoagulant surface on various experimental and medical devices.^10^ The strong anticoagulative effect of heparin is due to its inhibition of thrombin activity, which hinders the formation of fibrin and also suppresses platelet (PLT) adhesion, aggregation, and release.^11,12^ Therefore, heparin can cause the adverse events of bleeding and thrombocytopenia, especially in DLC patients and intensive care patients.^13^

In recent decades, normal saline (NS) has been globally advocated for the flushing and locking of PVC based on previous trials.^14,15^ However, in China, many infusion nurses continue to use conventional HS on a regular basis.^3^ Therefore, whether HS is more preferable for the flushing and locking of PVC than NS has been questioned. The present trial compared the safety and effectiveness of NS to those of HS for the flushing and locking of PVC in DLC patients and examined coagulation function and PLT function in these 2 patient groups.

## METHODS

### Eligibility

From April 2012 to March 2013, DLC patients admitted to the Department of Gastroenterology, West China Hospital, were considered for enrollment in this study. The inclusion criteria were as follows: diagnosis based on clinical manifestations, serologic tests, and imaging examinations; admission due to ascites, moderate–severe hepatic dysfunction, or endoscopic or interventional treatment; possible previous inpatient treatment history (considered acceptable); Child–Pugh grade A, B, or C; infusion treatment with therapeutic intent; age from 18 to 75 years, without gender limitation; and provision of informed consent. The exclusion criteria were as follows: infusion treatment within the past 30 days; massive upper gastrointestinal hemorrhage; severe hepatic encephalopathy; or obvious bleeding tendency.

### Randomization and Single-Blindness

The present trial was approved as a phase II clinical trial by the Biomedical Ethical Committee of West China Hospital, Sichuan University (serial number: 2013–114). Eligible patients who provided informed consent were randomly allocated to 2 groups, HS and NS. A simple randomization method was used, and random numbers were generated by MS Excel software. Allocation was concealed until the first blood sample was collected before infusion treatment, and the random numbers were managed by the principal investigator (WR). A single-blind approach was used, and the patients understood that they would not be informed of the exact type of flushing and locking fluid used.

### Infusion, Blood Sample, and Laboratory Results

Infusion procedures were performed by 2 standard-trained nurses (LO and HL) according to the Infusion Nursing Standards of Practice by the Infusion Nurses Society.^16^ In the HS group, HS (50 U/mL) was manually prepared. In the NS group, preservative-free 0.9% sodium chloride was used as the flushing and locking solution. In the 2 groups, the flushing and locking of PVC was required once or twice depending on the infusion situation, and 5 mL of the solution was flushed each time. Participants were blinded to which intervention they were receiving due to the similarity of the solutions. ZM, TL, and ZZ-X collected the patients’ information and also observed and recorded all adverse events. Fasting peripheral blood samples were collected twice from the upper limb that was contralateral to the infusion limb. The first specimen was collected before allocation and infusion, and the second specimen was collected at the day after infusion was completed. The specimens were immediately transferred to and tested in the Lab of Clinical Hemotology at West China Hospital. PLTs were counted in Ethylene Diamine Tetraacetic Acid (EDTA)-containing specimens (2 mL) using an XE-2100 flow cytometer. Prothrombin time (PT) and activated partial thromboplastin time (APTT) were tested in a sodium-citrate-containing specimen (3 mL) using a SYSMEX CA-7000 system. PLT, PT, and APTT were chosen as the 3 parameters for study because they can be directly affected by heparin.

### Data Collection and Outcome Measures

Data collection and management were performed by LJ-X using MS Excel software, and the data could not be accessed by the researchers. The patients’ baseline characteristics, including gender, age, Child–Pugh grade, anticoagulant administration, irritant agent administration, and PVC type, were recorded. The primary outcome measures included the maintenance time of each catheter and detailed reasons for catheter removal. The secondary outcome measures were PLT, PT, and APTT levels in peripheral blood.

### Statistics

The statistical analyses were performed after the observation had been completed by CX-Z using SPSS 13.0 software (SPSS, Inc., Chicago, USA), with randomization concealed from the analyzer. For continuous data with normal distributions, the one-way analysis of variance test was used, while for data without normal distributions, the Mann–Whitney *U* test was used. For categorical data, the Chi-square test or Fisher exact test was used. The Spearman correlation test was used for the univariate analyses. The Least-Significant Difference (LSD) post hoc test was used for multigroup comparisons. The PLT, PT, and APTT levels both pre- and postinfusion and the changes thereof were compared between the 2 groups. A 2-sided *P* value of less than 0.05 was considered to indicate statistical significance.

## RESULTS

### Patients

In this phase II trial, 68 eligible patients with adequate information were enrolled from April 2012 to March 2013 and included in the final analysis (36 and 32 patients in the HS and NS groups, respectively) (Supplementary Figure 1, http://links.lww.com/MD/A353). The cases excluded from the final analysis were those who rejected the collection of a second blood sample after infusion or had inadequate records. The baseline characteristics, including gender (*P* = 0.069), age (*P* = 0.241), Child–Pugh grade (*P* = 0.372), anticoagulant administration (*P* = 0.225), irritant agent administration (*P* = 0.218), and PVC type (*P* = 0.445), were generally comparable between the 2 groups (Table 1). Because the hospitalization stay and infusion duration were both longer in the NS group, the number of PVCs used in the HS and NS groups were different (65 versus 125 PVCs, respectively).

**TABLE 1:**
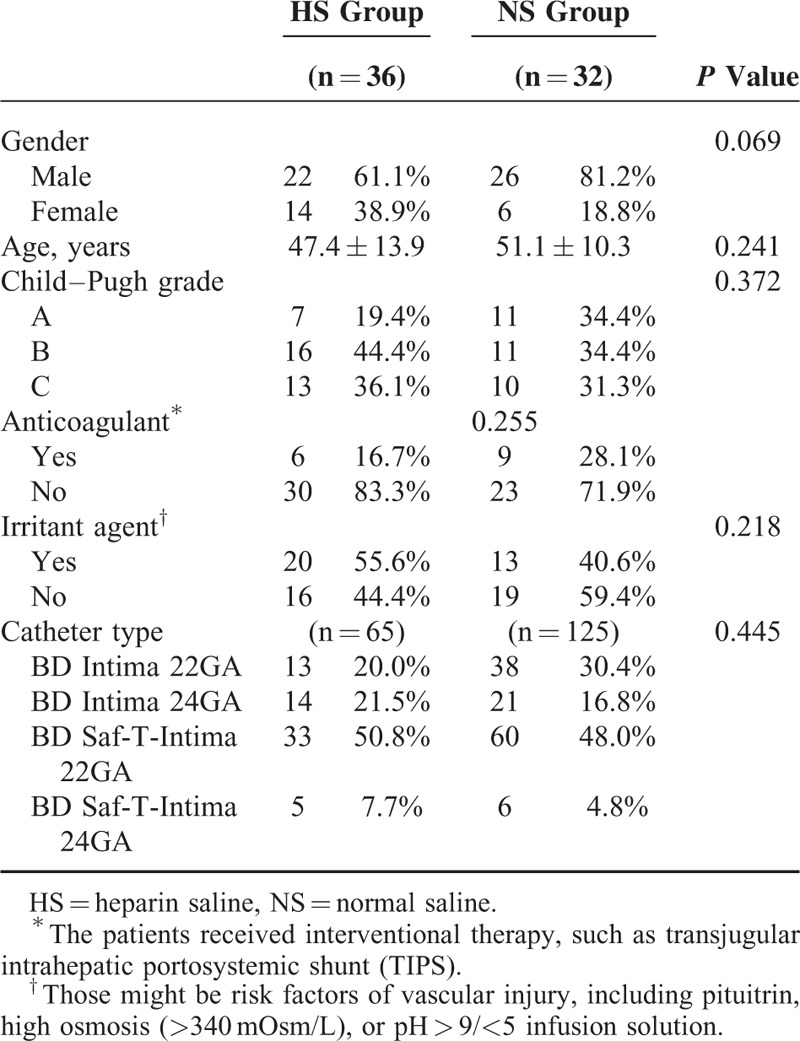
Baselines Comparison Between 2 Groups

### Maintenance of PVCs

The mean PVC maintenance times of the HS and NS groups were not significantly different, being 80.27 ± 26.47 hours and 84.19 ± 29.32 hours (*P* = 0.397), respectively (Table 2). The proportion of PVCs with maintenance times of more than 96 hours were 7.7% and 16.8% in the HS and NS groups, respectively (OR = 0.41, 95% CI 0.15–1.15, *P* = 0.083). Although there was no significant difference in the PVC maintenance duration between the 2 groups, a trend of longer maintenance duration in the NS group was observed to some extent.

**TABLE 2:**
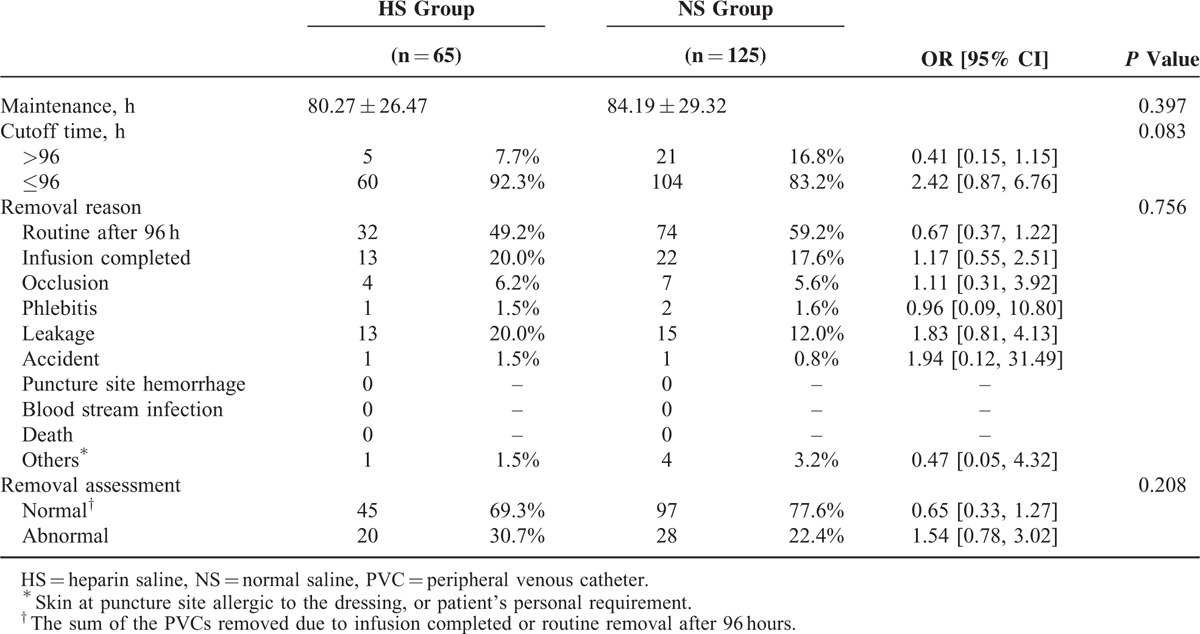
Maintenance and Removal Reasons of PVCs in 2 Groups

The proportion of PVCs removed for abnormal reasons were 30.7% and 22.4% in the HS and NS groups, respectively (OR = 0.65, 95% CI 0.33–1.27, *P* = 0.208), with an absolute risk increase of 8.3% in the HS group compared with the NS group; however, this difference was not statistically significant (Table 2). The catheter occlusion rates were 6.2% and 5.6% in the HS and NS groups, respectively (OR = 1.11, 95% CI 0.31–3.92). The most common adverse event resulting in abnormal removal was infusion leakage around the puncture site (20.0% versus 12.0%, OR = 1.82, 95% CI 0.81–4.13). Apparent phlebitis was rare in both the HS and NS groups (1.5% versus 1.6%, OR = 0.96, 95% CI 0.09–10.80). There was no case of puncture site hemorrhage, blood stream infection, or death.

### PT, APTT, and PLT Changes

**FIGURE 1:**
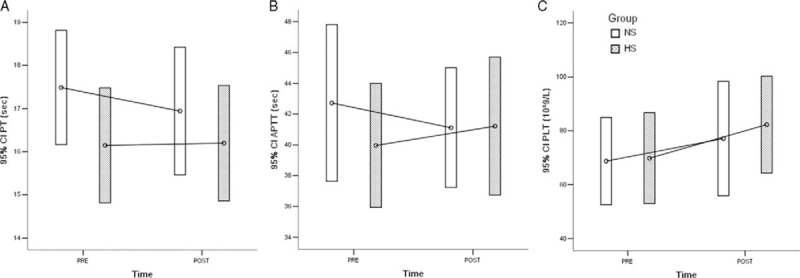
Error bar plots of preinfusion and postinfusion levels of (A) PT, (B) APTT, and (C) PLT in 2 groups. APTT = activated partial thromboplastin time, PLT = platelet, PT = prothrombin time.

First, the baseline levels of PT (*P* = 0.152), APTT (*P* = 0.392), and PLT (*P* = 0.926) before infusion were comparable between the 2 groups, but the mean levels of PT and PLT were apparently worse than the normal reference limits due to the presence of DLC (Table 3). The levels of PT (*P* = 0.453), APTT (*P* = 0.973), and PLT (*P* = 0.706) after infusion were also comparable between the 2 groups, without significant differences (Table 3). Interestingly, the PT and APTT levels were slightly worse after infusion than before infusion in the HS group, unlike in the NS group (Figure 1, Table 3). Moreover, in both groups, the PLT levels increased slightly from before to after infusion, but they remained below the lower limit (Figure 1, Table 3).

**TABLE 3:**
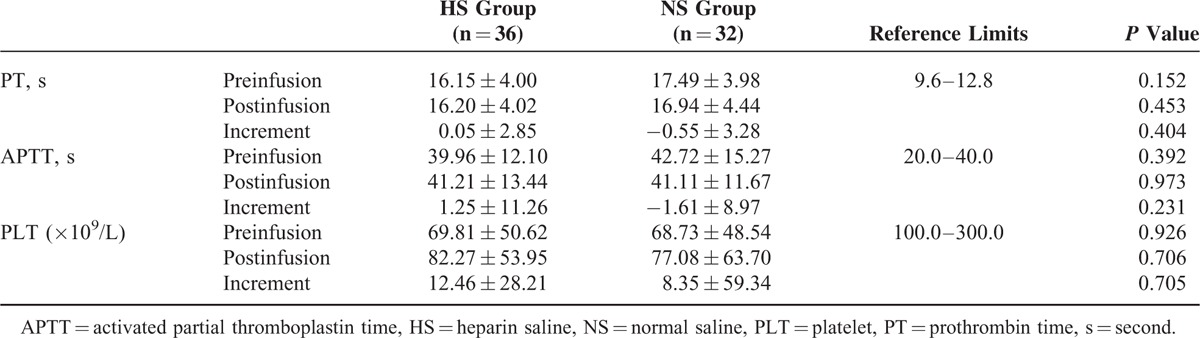
Comparisons on PT, APTT, and PLT Between 2 Groups

**FIGURE 2:**
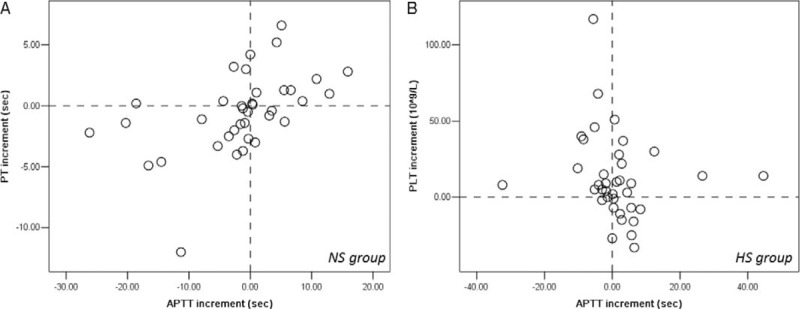
Scatter plots of correlations (A) between PT and APTT in NS group, and (B) between PLT and APTT in HS group. APTT = activated partial thromboplastin time, HS = heparin saline, NS = normal saline, PLT = platelet, PT = prothrombin time.

Therefore, further incremental analyses were performed to compare the dynamic changes in PT, APTT, and PLT levels; however, the increments of these levels were all comparable between the HS and NS groups, without significant differences (Table 3). The interactions between the dynamic changes in PT, APTT, and PLT levels were tested by correlation analyses (Table 4). In the NS group, there was a moderately positive and significant correlation between increments of PT and APTT (*r* = 0.607, *P* = 0.0002), while in the HS group, there was a mildly negative and significant correlation between increments of APTT and PLT (*r* = −0.380, *P* = 0.022) (Figure 2, Table 4). This finding implied that the improvement or deterioration of these parameters was potentially synchronous.

**TABLE 4:**
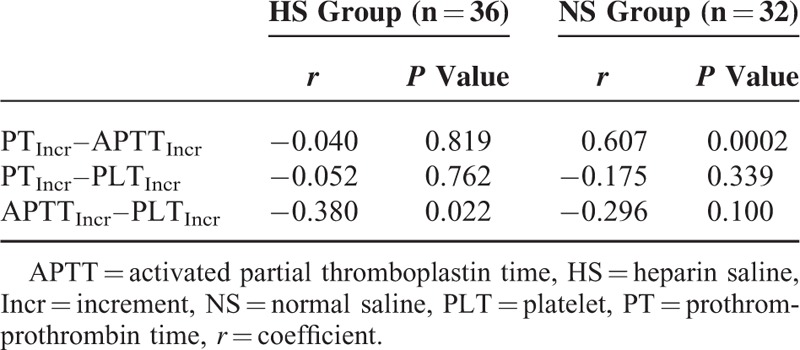
Interactive Correlations Among the Increments of PT, APTT, and PLT

**FIGURE 3:**
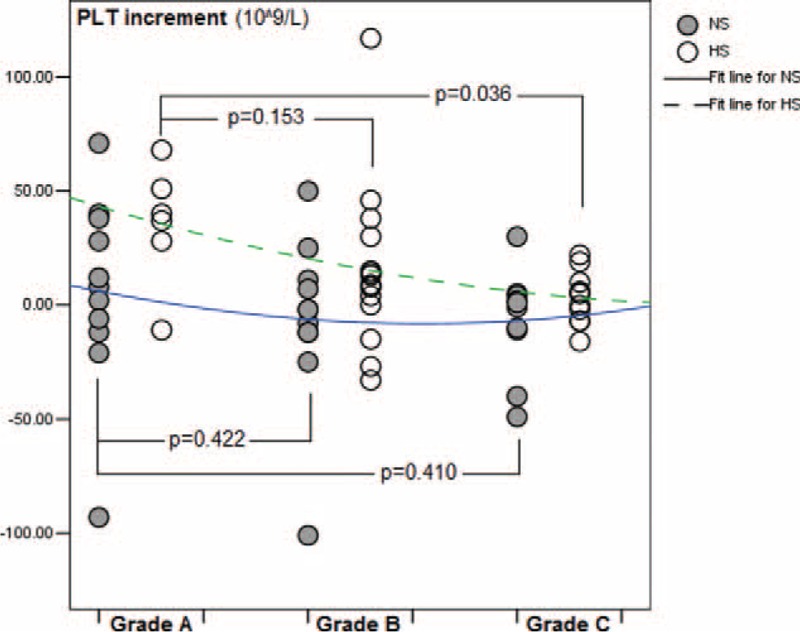
Child–Pugh grades as a risk factor on increment of platelet (PLT) count.

To identify certain potential risk factors that might influence the levels of PT, APTT, and PLT, univariate correlation analyses were performed in both the HS and NS groups. Gender, age, maintenance duration, PVC type, and anticoagulant or irritant agent administration were not relevant risk factors (Table 5). However, in the HS group, Child–Pugh grade was found to be a mild but significant risk factor of impairment of the normalization procedure of PLT count (*r* = −0.353, *P* = 0.035) (Table 5). Therefore, the increments of PLT levels among patients with Child–Pugh grade A, B, and C were compared to each other in both the HS and NS groups (Figure 3). The results indicated that in the HS group, the increase in the normalized PLT count in the Child–Pugh grade C subset was significantly suppressed compared with that of the Child–Pugh grade A subset (Figure 3).

**TABLE 5:**
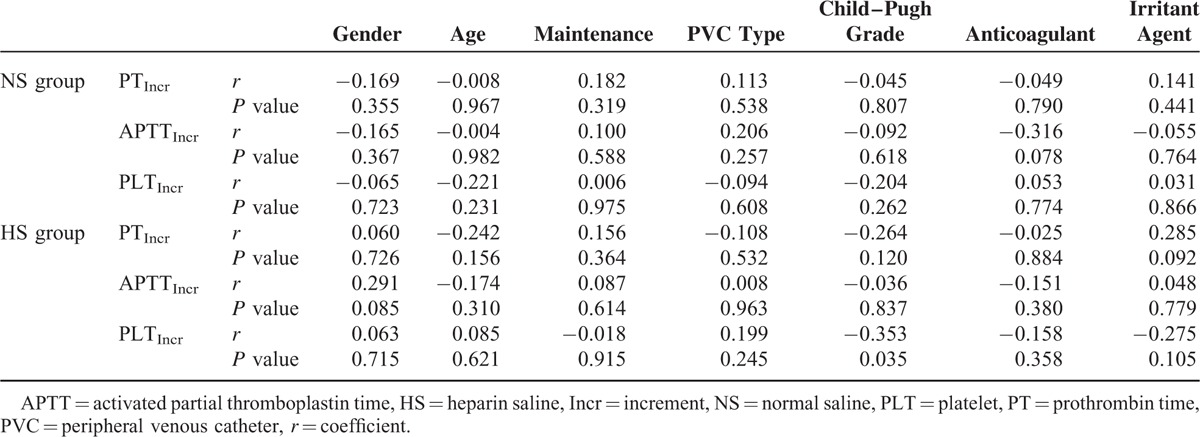
The Correlations Between Risk Factors and Increments of PT, APTT, and PLT

## DISCUSSION

The present randomized, controlled, single-blinded trial compared HS with NS for flushing and locking PVCs in DLC patients. The results showed that the maintenance duration, occlusion, phlebitis, and leakage were comparable between the 2 solutions; however, HS tended to mildly impair PT and APTT levels, especially in Child–Pugh grade C patients, although these impairments were not significant. This trial had some limitations that should be carefully discussed. First, as a phase II trial, the sample size was relatively small to obtain a confirmative conclusion, and trials of a larger scale are required to further confirm the present findings. Second, the rate of participant withdrawal was relatively high (19%, 16/84) in this trial due to refusal to participate after allocation. The impaired compliance in this study may have influenced the power of the results to a certain extent.

Since the 1980s, the necessity of HS for the flushing and locking of PVC has been questioned in Western countries.^17^ In 2011, the Infusion Nursing Standards of Practice prepared by the Infusion Nurses Society proposed the feasibility and safety of NS as a flushing and locking fluid for PVC.^16^ Compared with HS, NS is considered to have several advantages. First, it avoids the manual preparation of HS by nurses, which carries a risk of contamination that may result in blood stream infection for the patients and a risk of occupational exposure to accidental syringe needle sticks for the nurses. Thus, NS is helpful for enhancing infusion-related infection control. Moreover, from a pathophysiological perspective, NS also theoretically eliminates the potential risk of impairment to coagulation and PLT function induced by heparin for DLC patients.

However, the concept of using NS for PVC flushing and locking has been difficult to accept for Chinese infusion nurses. In China, conventional HS was preferred for PVC flushing and locking in common nursing practice until recently.^18^ To the best of our knowledge, relevant clinical evidence in the Chinese infusion nursing field on this subject is lacking, and as recently as 2005, most infusion nurses still regarded HS as more effective than NS, with high concentrations of HS being considered more effective than lower concentrations. Hence, many previous Chinese studies only focused on the influence of the different concentrations of HS on PVC flushing and locking. Although recent years have seen increasing attention being paid to NS for PVC flushing and locking in the Chinese infusion nursing field, a confirmative conclusion or domestic consensus in this area has not been achieved.

Our experiences in a previous prospective, controlled trial in 2011 also supported the feasibility and safety of NS for PVC flushing and locking.^3^ This previous trial included patients with gastroenterology and hepatic diseases, with 178 and 181 patients in the HS and NS groups, respectively. The results demonstrated that NS neither shortened the duration of PVC maintenance (3.6 ± 1.1 days versus 3.7 ± 1.2 days, *P* = 0.651) nor increased the proportion of abnormal withdrawal (29.3% versus 31.5%, *P* = 0.654). Furthermore, there was no difference in the occlusion rates between the 2 groups.^3^

The liver is the location of coagulation factor synthesis. The abnormality of coagulation factors in cirrhosis patients aggravates the disease, resulting in poor coagulation status and bleeding tendency.^8,19^ The coagulation time of cirrhosis patients is obviously prolonged: PT, APTT, thrombin time (TT), and fibrin (Fib) levels all change to some extent, with PT, TT, and APTT being significantly prolonged and Fib being significantly decreased.^9,20^ Along with the deterioration of cirrhosis, liver function and coagulation function can progressively worse. Hypersplenism is subsequent to DLC and can cause a decrease in PLT count.^21,22^ Therefore, both PLT count and coagulation parameters have abnormal changes and can be highly associated with Child–Pugh grades. Thus, it is particularly important to accurately assess the degree of liver dysfunction to predict bleeding tendency.

Heparin, a highly sulfated glycosaminoglycan, is a common anticoagulant that has a strong anticoagulation effect both in vivo and in vitro. Heparin primarily acts by enhancing the affinity of antithrombin-III (AT-III) to thrombin and resulting in the acceleration of the inactivation of thrombin.^23,24^ Low-dose heparin has been commonly administered to prevent venous thrombosis with satisfying effectiveness and safety and also with minor interference in laboratory examinations; therefore, heparin has been used to flush and lock PVCs to maintain their patency.^25,26^ However, DLC patients have coagulation dysfunction with higher bleeding tendency; therefore, HS for the maintenance of PVC is controversial in these patients due to the potential risk of further impairment. Moreover, any form of heparin is able to induce thrombocytopenia, probably through an immunological mechanism.^27^ Research has shown that high-molecular-weight heparin is prone to interact with PLTs to induce thrombocytopenia, and this finding was in accordance with the clinical observation that patients treated with low-molecular-weight heparin had a lower incidence of thrombocytopenia.^28,29^ In a Chinese observational study, there were 14 (1.2%) cases of heparin-induced thrombocytopenia out of 1215 patients given heparin to flush and lock PVCs.^30^

In summary, the present randomized, controlled, single-blinded trial compared HS with NS for the flushing and locking of PVCs in DLC patients, and the results demonstrated that the maintenance duration, occlusion, phlebitis, and leakage were comparable between the 2 solutions. Additionally, despite the lack of significance, HS tended to mildly impair the PT and APTT levels in these patients and should especially be avoided in Child–Pugh grade C patients. Therefore, we concluded that NS may be as effective as and safer than HS for the flushing and locking of PVCs in DLC patients.
